# Central precocious puberty associated with duplicated pituitary: a case report and literature review

**DOI:** 10.3389/fendo.2025.1466411

**Published:** 2025-02-11

**Authors:** Kun Yang, Yanmei Sang, Li Dai, Weizhong Wang

**Affiliations:** ^1^ Baoding Hospital, Beijing Children’s Hospital affiliated to Capital Medical University, Baoding, Hebei, China; ^2^ Department of Endocrinology, Genetics and Metabolism, Beijing Children’s Hospital, National Center for Children’s Health, Capital Medical University, Beijing, China; ^3^ Beijing Key Laboratory for Genetics of Birth Defects, Beijing, China

**Keywords:** GnRHa, central precocious puberty (CPP), duplicated pituitary, female, height

## Abstract

Patients with duplicated pituitary often have severe neurodevelopmental abnormalities and craniofacial deformities. Till now, only eight patients diagnosed with duplicated pituitary showing barely central precocious puberty (CPP) are reported in the published literature. Herein, we report a 6-year-old and 3-month-old girl diagnosed with pituitary duplication presented with CPP. She has enlarged breasts for more than 1 year, accelerated height of linear growth, advanced bone age, increased hormone level, and increased volume of uterus and ovary. A duplicated pituitary gland was shown in the MRI. As the gonadotropin-releasing hormone analogue (GnRHa) stimulation test showed a positive result, the child was treated with GnRHa. After 2 years of follow-up, there was a delay in the progression of sexual development, a reduction in symptoms of precocious puberty, and an increase in predicted adult height.

## 1 Introduction

Central precocious puberty (CPP) is a common pediatric disease that results in the rapid development of internal and external reproductive organs and secondary sexual characteristics before the age of 7.5 in girls and 9 in boys due to the early activation of the hypothalamus–pituitary–gonad axis (1). The etiology of CPP is complex and includes congenital central nervous system diseases such as hamartoma, optic chiasmatic glioma, pineal tumor, and pituitary duplication. Among them, CPP caused by pituitary duplication is extremely rare, with a total of eight cases found (2–8). Pituitary duplication is a very rare developmental disorder, the prevalence of which remains unclear. It was proposed that the development disorder leading to duplication may have caused precocious secretion of luteinizing hormone-releasing hormone as a consequence of nuclear derangement and failure of regulation (2–4). Published studies reported the main clinical manifestations and comorbidities, with little illustration of height change. In this study, we report a patient diagnosed with CPP resulting from pituitary duplication, with a detailed description above height changes, and make a literature review in CPP associated with duplicated pituitary.

## 2 Case report

A 6-year-old and 3-month-old girl presented to our hospital with precocious puberty due to enlarged breasts for more than a year. Her height was 121 cm (+0.65 SD, P71.6) (9), weight 24.6 kg (+1.39 SD, P87.4) (9), and BMI 16.8 kg/m^2^. Her breasts are in Tanner III, her pubic hair is in Tanner I, and she has no armpit hair. No abnormality was found in the cardiopulmonary abdominal examination and nervous system examination. No anosmia was observed. The child was G2P2, with no abnormal obstetric history. Her birth weight was 3.6 kg, her height 50 cm. She had no consanguineous marriage and family history of hereditary diseases. The height of her father is 170 cm, and the age of puberty initiation is unknown; the height of her mother is 168 cm, and menstrual menarche was 14. Her brother is 14 years old, with height 170 cm.

The following are the test results: adrenocorticotropin (ACTH) 25 pg/ml (0 pg/ml–45 pg/ml), cortisol (F) 111 nmol/L (96.55–551.72 nmol/L), basal hormone level (with immunochemiluminescence assays/ICMA): FSH 5.62 IU/L (0.11 U/L–1.60I U/L), LH 0.98 IU/L (<0.1IU/L), prolactin (PRL) 7.58 ng/ml (2.0 ng/ml–43 ng/ml), estradiol (E2) 88.88 pg/ml (0 ng/ml–31 pg/ml), testosterone (T) < 2.5 ng/dl (0.00 ng/dl–32.3 ng/dl), progesterone (P) 0.05 ng/ml (0.00–1.40 ng/ml). The GnRHa stimulation test showed a positive result, with the LH peak > 5.0 IU/L and the LH peak/FSH peak > 0.6.

The breast ultrasound examination results showed the development of her bilateral breast. Ultrasound results showed 3.7 cm*0.9 cm*0.8 cm of the uterus, 2.9 cm*0.9 cm of the left ovary, four follicles of 0.4 cm*0.4 cm and above in diameter, 3.4 cm*1.1 cm of the right ovary, and four follicles of 0.4cm*0.4cm and above in diameter. Ultrasound results of the adrenal gland showed no abnormality. The bone age was 8 years old.

The girl was diagnosed with central precocious puberty according to the following diagnostic criteria: early onset of sexual characteristics, with breast development occurring before the age of 7.5 years; pelvic ultrasound showed that the girl’s uterus and ovaries were enlarged, and multiple follicles ≥4 mm in diameter were seen in the ovaries; serum gonadotropins and sex hormones reach pubertal levels, peak LH ≥5.0 IU/L and LH peak/FSH peak> 0.6, suggesting initiation of the gonadal axis; bone age ≥1 year above actual age; accelerated linear growth (1).

**Figure 1:**
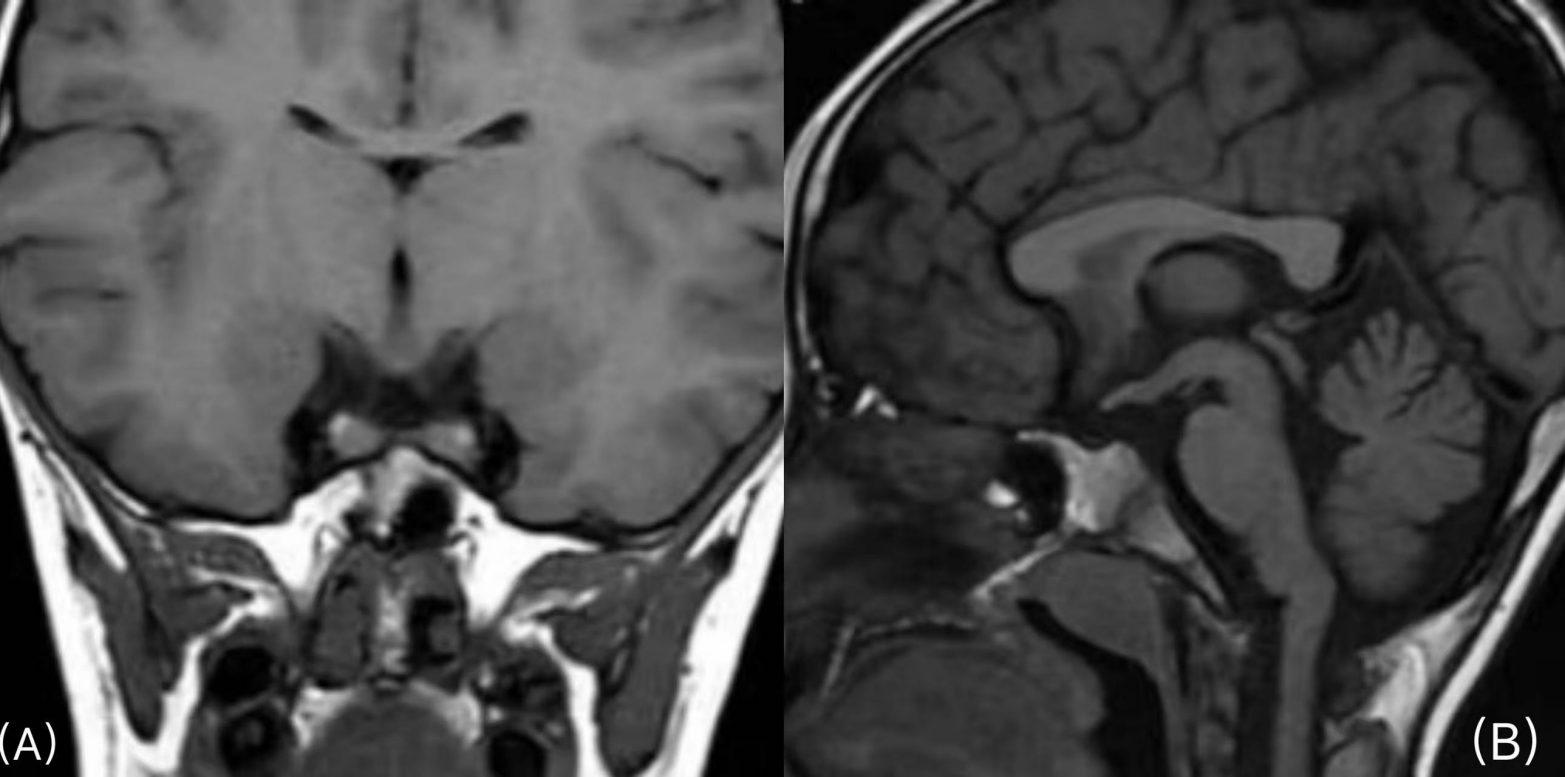
**(A)** Coronal plane scan of the pituitary gland: one pituitary gland is seen on each side of the sella turcica, and the two pituitary stalks walk in the shape of “八” (a Chinese character). **(B)**, Sagittal scan of the pituitary gland: the shape of the hypothalamus is irregular, and the center of sella turcica becomes shallower.

Magnetic resonance imaging of the brain showed pituitary duplication (one pituitary on each side of sella turcica, approximately 4.9 mm on the left and approximately 5.5 mm on the right), and there seemed to be a thin film structure connected between them. No abnormal signal was found in the pituitary, a high signal of neuro pituitary T1W1 existed, and the hypothalamus thickened. The coronal position shows two pituitary stalks in a “八” shape, and the center of sella turcica became shallower (**Figure 1**).

Her adult height was predicted to be 153.3 cm; she was not receiving treatment at the time of diagnosis. Nine months later, her LH was higher than before (2.24 IU/L). Pelvic ultrasound showed that the volumes of her uterus and ovary were larger than before (4.4 cm*1.6 cm*1.0 cm of the uterus, 2.8 cm*1.1 cm of the left ovary, five follicles of 0.4 cm*0.4 cm and above in diameter; 3.5 cm*1.12 cm of the right ovary, six follicles of 0.4 cm*0.4 cm and above in diameter). Her bone age was 9 years old. All available evidence points to the conclusion that the patient was suffering from rapidly progressive central precocious puberty. She started receiving treatment at age 7; leuprorelin microspheres of 3.75 mg were subcutaneously injected once every 28 days for more than 2 years. After that, she showed breast retraction, reduction of pubic hair and axillary hair, and a decline in sex hormones (such as LH and FSH). The ultrasound indicated that uterine volume, ovarian volume, bilateral ovarian diameter 0.4 cm*0.4 cm, and number of follicles have decreased; her bone age slowed down. At the age of 8, she was 134.9 cm (+1.18 SD, P83.4) (9) in height and 34.8 kg (+2.30 SD, P96.5) (9) in weight. At the age of 9 years and 3 months, she was 143.5 cm (+1.26 SD, P86.1) (9) in height and 32.9 kg (+0.83 SD, P71) (9) in weight. The fluctuations in hormone levels before and after treatment are illustrated in **Table 1**.

**Table 1:** Changes in hormones.

	6 years and 3 months	7 years	7 years and 3 months	8 years	9 years
Follicle-stimulating hormone (FSH) IU/L	5.62	5.85	1.63	2.15	3.06
Luteinizing hormone (LH) IU/L	0.98	2.24	0.29	0.23	0.19
Prolactin ng/ml	7.58	7.38	13.17	10.16	11.02
Estradiol (E2) pg/ml	88.88	80.66	<5	<5	<5
Testosterone ng/dl	<2.5	10.91	<2.5	<2.5	<2.5
Progesterone ng/ml	0.05	0.15	0.12	0.05	0.05

## 3 Discussion

In this study, the female patient presented with precocious puberty, which is characterized by enlarged breasts, accelerated linear height growth, increased bone age, positive gonadorelin provocation test result, increased utero-ovarian volume, and increased number of large follicles. The MRI results revealed pituitary duplication. She was diagnosed with central precocious puberty associated with duplicated pituitary. The treatment with GnRHa had been ongoing for over 2 years, with a favorable prognosis. During the follow-up visit before submission, it was confirmed that the patient was 12 years old, with a height of 158 cm, and had been menstruating for 1 year.

Pituitary duplication is an extremely rare phenomenon. It was first discovered and reported by Ahlfeld in 1880 (7). In 2000, Burke M proposed that pituitary duplication may lead to the early activation of the hypothalamus–pituitary–gonadal axis (2).

The formation of the hypophysis depends on the interaction of the embryonic primordium with normal growth processes in the prechordal region of the head (4). The prechordal plate and the rostral portion of the notochord are closely related to the development of the pituitary gland [2.4.10]. The interaction among pituitary tissues is regulated by multiple transcription factors, which are regulated by many genes, such as HESX1 (homeobox gene expressed in ES cells1), LHX4 (Lim homeobox4), PROP1 (homeobox protein prophet of Pit1), and SOX (SRY-related HMG-box) (10–13). It is speculated that the duplication of the rostral end of the notochord may act as the main factor that leads to duplication of the pituitary primordium, which may be caused by smoking and drugs in early pregnancy, with resultant formation of two morphologically normal glands, skull base malformation, and midline malformation [4.8.14].

21% of patients with pituitary duplication present with dysfunction of the hypothalamus–pituitary–gonad axis (7). The majority (approximately 73%) of patients presenting with a hypothalamus–pituitary–gonad axis showed precocious puberty, and a small number (approximately 27%) showed delayed puberty or hypogonadism (7).

Eight patients diagnosed with pituitary duplication related to precocious puberty were all found to have hypothalamic malformation. Hypothalamic thickening is the most common anatomical abnormality, and it may result in precocious or delayed puberty because of blocked lateral movement of hypothalamic nucleic cells from nuclear deregulation and dysregulation (4). Among the eight cases, four cases present with facial deformities, three cases with vertebral malformations, three cases with basilar artery duplication, two cases with cleft palate, and one with anosmia. Seven children were treated with GnRHa, and the another one with a height of 156 cm at the age of 8.5 years old was not treated with GnRHa. More data is shown in **Table 2**. In this study, the girl’s breast development began at 5 years and 3 months old. At 6 years and 3 months old, there was an increase in the volume of the uterus and ovary, and the number of 0.4 cm*0.4 cm ovaries in the ovary. The bone age was 8 years old. The GnRHa stimulation test showed a positive result. All the results above met the diagnostic criteria of CPP (1). The clinical manifestation of the child is precocious puberty. MRI results showed pituitary duplication, hypothalamus thickening, and no cervical vertebra deformity. There was no craniofacial deformity, loss of smell, or other manifestations. It was a mild case of duplicated pituitary. The clinical and pituitary MRI findings of the case reported by Burke in 2000 are consistent with this study (2). Burke suggests a different mechanism to explain isolated pituitary duplication: a primary disruption in the area of the neuroectodermal adhesion, independent of the notochord, would result in disjunction of the primordium and, consequently, hypophyseal duplication (2).

**Table 2:** Eight patients with duplicated pituitary and precocious puberty.

	Burke et al. (2)	Shroff et al. (3)	de Penna et al. (4)	de Penna et al. (4)	Vieira et al. (5)	Krnic et al. (6)	Leyla Akin et al. (7)	P Spiller et al. (8)
Year	2000	2003	2005	2005	2007	2009	2011	2020
Country	USA	Canada	Brazil	Croatia	Brazil	Croatia	Turkey	USA
Literature source	Pediatr.Neurosurg	AmJNeuroradiol	Arg. Bras. Endocrinol Metabol.	Arg. Bras. Endocrinol Metabol	J PediatrEndocrinolMetab	LijecVjesn	Childs Nerv Syst	Int J PediatrOtorhinolaryngol
Puberty development	Regular menstrual cycle at 8.5 years old	Unknown	Breast development begins at age 6	Breast development begins at 6 years and 8 months old	Menstrual onset at age 7	Breast development begins at age, menstrual onset at age 7	Breast development begins at the age of 7	Breast development begins at 4.5 years old
Hypothalamic malformation	+	+	+	+	+	+	+	+
Basilar artery duplication	–	+	–	–	–	+	–	+
Vertebral malformations	–	–	–	–	+	–	+	+
Cleft palate	–	–	–	+	–	–	–	+
Other complications	Absent olfactory bulb/tracts	Mandible duplication, teratoma	Micrognathia, rhinoschitis, oropharyngeal masses	Uvula and tooth abnormalities, esophageal fistula	None	Nasal deformities, upper lip fibro-lymphoma	None	None
Treatment	None	GnRHa	GnRHa	GnRHa	GnRHa	GnRHa	GnRHa	GnRHa

(+, present; -, not present).

In contrast, three patients diagnosed with pituitary duplication in previous reports showed delayed puberty and hypogonadotropic hypogonadism. In 1995, S.S. Kollias in the United States reported a 16-year-old girl with delayed treatment due to puberty, accompanied by oropharyngeal masses, without short stature or anosmia (14). In 2010, Gu Changwei reported a 19-year-old girl diagnosed with pituitary dwarfism/pituitary dysfunction. At the age of 12, the patient was diagnosed with growth retardation, no secondary sexual development, decreased visual sensitivity, and no loss of olfactory sensation. MRI results suggested a cystic abnormal signal in the sellar region, extension of the third ventricle and extension into the sphenoid sinus, no obvious enhanced pituitary signal, absence of the sellar floor of the sellar boot, and no indication of the anterior commissure. The pituitary structure can be seen on both sides of sella turcica (15). In 2020, Asma Ahmed of Pakistan reported a 28-year-old adult man of Asian descent, diagnosed as hypogonadism with hypogonadism, infertility, dermoid cyst, cervical vertebra deformity, and loss of olfaction (16). The results above suggest that in the cases of pituitary duplication, the clinical symptoms are different in severity and individualized.

Neuroectodermal disjunction may occur as a primary event leading to isolated pituitary duplication or secondary to prechordal plate anomalies in which the duplication would be associated with other craniofacial malformations (2), such as facial deformities, abnormal tongue development, cleft lip and palate, hypoplasia of corpus callosum, and nasopharynx mass [3.19].

**Figure 2:**
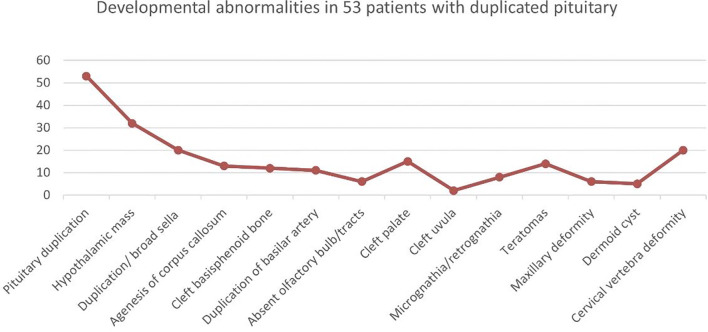
Developmental abnormalities in 53 patients with duplicated pituitary.

We identified a total of 53 patients (including adults) with pituitary duplication. Hypothalamic malformations were the most common, followed by enlarged sphenoid bone, cervical vertebra fissure, cleft palate, and teratoma (nasopharyngeal mass), as shown in **Figure 2**. Those of adults with pituitary duplication (except autopsy-reported cases) were relatively mild, mostly due to nasopharyngeal mass, upper limb numbness, etc., and craniofacial deformities were not obvious (17–20).

Most cases can be diagnosed in the neonatal period. Most of them died in infancy because of severe craniofacial deformities. Until 2015, only eight patients with pituitary duplications survived beyond puberty (16). Most of these cases that survived after puberty were accidentally found because of the abnormal process of puberty (16). The patient presented only with precocious puberty and was responsive to GnRHa treatment. We hypothesized that the prognosis for mild patients might be favorable. However, the later progress still needs long-term follow-up and observation.

Treatment strategies for the disease were formulated based on symptoms. GnRHa is effective in patients with central precocious puberty. Of the eight cases of pituitary duplication with precocious puberty, seven cases were treated with GnRHa, and the sexual development was effectively delayed. In this report, the child was treated with leuprorelin at the age of 7 years. After follow-up for more than 2 years, the sexual development was delayed and predicted adult height was improved.

All patients with craniofacial deformities generally need surgical treatment, and a detailed preoperative evaluation and comprehensive treatment plan should be made. Palatoplasty is necessary for cleft palate (21). Most of the oropharyngeal masses are hamartomas or teratomas. Hamartomas are benign lesions and can be treated conservatively (19). Those with teratomas that compress the airway must be removed surgically (21). In the case of mandibular duplication, the redundant mandibular tissue should be removed from the lingual adhesion area, and in the case of tongue duplication, the redundant tongue tissue should be removed for tongue reconstruction (22, 23).

In brief, the clinical manifestations of pituitary duplication vary greatly in severity, with most cases accompanied by severe neurodevelopmental disorders and craniofacial deformities. The combination of isolated pituitary duplication and precocious puberty is extremely rare in clinical settings, and the therapeutic efficacy of inhibitors is favorable. However, the prognosis of most of these patients is unfavorable. Hence, long-term and meticulous follow-up is necessary to promptly detect relevant complications and address the manifestations in time, effectively improving the prognosis of patients.

## Data Availability

The original contributions presented in the study are included in the article/supplementary material. Further inquiries can be directed to the corresponding authors.
